# Neuroprotective Effect of *Caryocar brasiliense* Camb. Leaves Is Associated with Anticholinesterase and Antioxidant Properties

**DOI:** 10.1155/2018/9842908

**Published:** 2018-10-21

**Authors:** Thiago Sardinha de Oliveira, Douglas Vieira Thomaz, Hiasmin Franciely da Silva Neri, Letícia Bonancio Cerqueira, Luane Ferreira Garcia, Henric Pietro Vicente Gil, Roberto Pontarolo, Francinete Ramos Campos, Elson Alves Costa, Fernanda Cristina Alcantara dos Santos, Eric de Souza Gil, Paulo César Ghedini

**Affiliations:** ^1^Institute of Biological Sciences, Federal University of Goiás, Goiânia, Brazil; ^2^Faculty of Pharmacy, Federal University of Goiás, Goiânia, Brazil; ^3^Department of Pharmacy, Federal University of Paraná, Curitiba, Brazil; ^4^Federal Institute of Goiás, Trindade, Brazil

## Abstract

Pequi (*Caryocar brasiliense*) is an endemic species from Brazilian Cerrado, and their fruits are widely used in regional cuisine. In this work, a crude hydroalcoholic extract (CHE) of *C. brasiliense* leaves and its resulting fractions in hexane (HF), chloroform (CF), ethyl acetate (EAF), and butanol (BF) were investigated for their antioxidant properties and anticholinesterase activities. The antioxidant properties were evaluated by free radical scavenging and electroanalytical assays, which were further correlated with the total phenolic content and LC-MS results. The acetylcholinesterase and butyrylcholinesterase inhibitory activities were examined using Ellman's colorimetric method. The LC-MS analysis of EAF revealed the presence of gallic acid and quercetin. CHE and its fractions, EAF and BF, showed anticholinesterase and antioxidant activities, suggesting the association of both effects with the phenolic content. In addition, behavioral tests performed with CHE (10, 100, and 300 mg/kg) showed that it prevented mice memory impairment which resulted from aluminium intake. Moreover, CHE inhibited brain lipid peroxidation and acetyl and butyryl-cholinesterase activities and the extract's neuroprotective effect was reflected at the microscopic level. Therefore, the leaves of pequi are a potential source of phenolic antioxidants and can be potentially used in treatments of memory dysfunctions, such as those associated with neurodegenerative disorders.

## 1. Introduction

Neurodegenerative disorders, such as Alzheimer's disease (AD), present features of memory and cognitive impairment. Literature reports that AD is characterized by a combined loss of cholinergic neurons and their projections to the basal nucleus and associated areas of the brain stream. Given that neurotransmission is nonetheless sensitive to beta-amyloid peptide toxicity, the progressive deterioration of cholinergic innervation leads to signaling impairment, therefore contributing to the cognitive and behavioral dysfunctions seen in AD [1, 2].

Oxidative damage is known to play an important role in neuronal damage, due to the neurodegeneration promoted by highly reactive compounds. Since brain tissue is particularly sensitive to reactive oxygen species- (ROS-) mediated cell damage, ROS build up may lead to lipid peroxidation. This process inhibits henceforth neurotransmitter production, such as that of acetylcholine, which is deeply involved in memory and learning [3, 4].

Considering the overall aspects of AD pathogenesis, the identification of antioxidant bioactive compounds presenting complementary anticholinesterase (AChE) activities is an important step to aid neuroprotector treatments, due to the possibility of synergistically mopping up ROS and allowing proper acetylcholine build up in the synaptic cleft. In this context, Brazilian Cerrado trees are known to possess myriads of antioxidant secondary metabolites which may also hinder different forms of cholinesterase enzymes [5, 6].

Amongst potential neuroprotective phytomedicines is *Caryocar brasiliense* (Camb), a Caryocaraceae family member popularly known as “pequi.” This tree plays a significant role in central western Brazilian culture and is a source of raw material for small- and middle-sized industries. In folk medicine, the fruit pulp is used as stomachic and for flu treatment, whereas a decoction of the leaves and flowers is used as energetic, tonic, aphrodisiac, and treatment for liver diseases. *C. brasiliense* is also known to be rich in flavonoids which may display leishmanicidal, antifungal, antioxidant, and vasorelaxant properties [7–10].

Studies concerning *C. brasiliense* leaves reported the presence of antioxidant compounds such as gallic acid, quinic acid, quercetin, and quercetin 3-o-arabinose [9, 10]. However, literature reports on the extent of neuroprotection related to *C. brasiliense* extract ingestion, as well as anticholinesterase activities, are still limited [11].

This study therefore is aimed at evaluating the antioxidant and anticholinesterase activities as well as neuroprotective effects of *C. brasiliense* leaves, in order to provide new information on the potential use of this plant against neurodegenerative disorders.

## 2. Material and Methods

### 2.1. Reagents

Bovine serum albumin, gallic acid, rutin and quercetin, aluminium chloride, acetylcholine, 5,5′-dithiobis(2-nitrobenzoic acid) (DNTB), 1,5-bis(4-allyldimethylammoniumphenyl)pentan-3-one dibromide (BW284c51), tetraisopropyl pyrophosphoramide (iso-OMPA), acetylthiocholine iodide, S-butyrylthiocholine iodide, epinephrine bitartrate, and eosin were purchased from Sigma.

This work also made use of hydrogen peroxide (CRQ), trichloroacetic acid (TCA) (Vetec), thiobarbituric acid (TBA) (TediaBrasil), n-butanol (Synth), glycine (Vetec), NaH_2_PO_4_H_2_O (Cromoline), Na_2_HPO_4_ (Synth), coomassie brilliant blue (Amresco), ethyl alcohol (Synth), xylene (Vetec), Paraplast (Merck), and hematoxylin (Merck).

All electrolyte salts, solvents and reagents were of analytical grade. Electrolyte solutions were prepared with double distilled Milli-Q water (conductivity ≤ 0.1 *μ*S·cm^−1^) (Millipore S. A., Molsheim, France).

### 2.2. Animals

Male Swiss mice (25–30 g) from the colony of the Federal University of Goiás were used in this study. The animals were housed under a controlled 12 h light/dark cycle and stable temperature (22-23°C) with free access to food and water. All experiments were conducted in accordance with the Sociedade Brasileira de Ciência em Animais de Laboratório (SBCAL) and were approved by the local Ethics in Research Committee (protocol number: 140/10).

### 2.3. Extract Preparation

*C. brasiliense* dried leaves were collected in September 2010 in Gurupi, Tocantins, Brazil. The plant was authenticated by Professor Aristônio Magalhães Teles, and a voucher sample was deposited in the herbarium of the Institute of Biological Sciences of the Federal University of Goiás under the code 1353 [10].

The extract was prepared focusing on polyphenol extraction; henceforth, CHE was obtained by immersion and sonication of leaf powder in ethanol-water solution (70 : 30) for 1 h. The resulting extract was lyophilized and stored in a dark container at 4°C.

Organic fractions (OFs) were obtained by fractionation of crude hydroalcoholic lyophilized extract using organic solvents with crescent degrees of polarity (hexane—chloroform—ethyl acetate—butanol). Thereafter, solvents were evaporated under reduced pressure to produce a hexane fraction (HF), a chloroform fraction (CF), an ethyl acetate fraction (EAF), and a butanol fraction (BF).

### 2.4. Determination of In Vitro Anticholinesterase Activity

Acetylcholinesterase (AChE) and butyrylcholinesterase (BChE) inhibitory activities were measured by the spectrophotometric method developed by Ellman et al. [12]. AChE and BChE from whole mice brain homogenates were used, while acetylthiocholine iodide (ATC) and butyrylthiocholine chloride (BTC) were employed as substrates of the reaction. In order to distinguish if pequi extracts had preferential action between AChE and BChE, tests were performed using ATC as a substrate in the presence of iso-OMPA (an inhibitor of BChE).

DTNB was used for the measurement of anticholinesterase activity. CHE and OFs (1–500 *μ*g/mL) were preincubated with the homogenates for 30 min before addition of DTNB and ATC or BTC. Neostigmine was used as a reference compound.

### 2.5. Antioxidant Assays

#### 2.5.1. DPPH Free Radical Scavenging Assay

The free radical scavenging activity was determined spectrophotometrically by reaction with 2,2-diphenyl-1-picrylhydrazyl (DPPH), as described by Blois [13]. CHE and OFs (1–500 *μ*g/mL) were preincubated for 30 min in the presence of DPPH before spectrophotometric analysis. Quercetin was used as an antioxidant standard.

#### 2.5.2. Voltammetric Determination of Redox Behavior

The electroanalytical profile of CHE and OFs was performed according to the method described by Lino et al. [14]. The electrochemical analysis was performed in phosphate buffer 0.1 M (pH 7.0). Voltammetric experiments were carried out using a potentiostat/galvanostat μAutolab III® integrated to the GPES 4.9® software (Eco Chemie, Utrecht, Netherlands).

Measurements were carried out in a 5 mL one-compartment electrochemical cell, using a three-electrode system consisting of a glassy carbon electrode (GCE) (*Ø* = 2 mm) as the working electrode, Pt wire as the counter electrode, and Ag/AgCl (3 mol·L^−1^ KCl) as the reference electrode. The experimental conditions for differential pulse voltammetry (DPV) and square wave voltammetry (SWV) were a pulse amplitude of 50 mV, pulse width of 0.4 s, and scan rate of 5 mV·s^−1^.

Measurements of pH were carried out in a QUIMIS® pH meter. All experiments were done at room temperature. All voltammograms presented were background subtracted and baseline corrected using the moving average application with a step window of 2 mV included in the GPES software herein employed. This mathematical treatment improves the visualization and the identification of peaks over the baseline without introducing any artifacts, even though the peak intensity is in some cases reduced (<10%) in relation to the untreated curve. Nevertheless, this mathematical treatment was used in the presentation of all experimental voltammograms for a better and clearer identification of the peaks. The values for the peak current presented in all plots were determined from the original untreated voltammograms after subtraction of the baseline.

The antioxidant capacity was presented by electrochemical index (EI), obtained by index *I*_pa_/*E*_pa_, using the mathematical calculation:

(1) $$\begin{array}{c} EI=\frac{I_{pa_{1}}}{E_{pa_{1}}}+\frac{I_{pa2}}{E_{pa2}}+\cdots +\frac{I_{{pa}_{n}}}{E_{{pa}_{n}}}. \end{array}$$

#### 2.5.3. Phenolic Content

The total phenolic content was estimated using the Folin-Ciocalteu reaction. Briefly, 2.5 mL of diluted Folin-Ciocalteu reagent (1/10) was added to a small volume of sample (usually between 25 and 100 *μ*L), which was then treated with sodium carbonate solution as described in Georgé et al. [15]. The absorbance was measured at 760 nm, and the total phenolic content was calculated as a gallic acid equivalent (GAE) based on a standard curve of gallic acid. All of the experiments were performed in triplicate. Results were expressed as milligrams of GAE/mL in both CHE and OFs.

### 2.6. MS Analysis

#### 2.6.1. Standards and EAF Preparation for LC-MS Analysis

Stock solutions of gallic acid and quercetin standards were prepared separately in methanol at concentrations of 1 mg/mL. All stock solutions were stored under refrigeration at 4°C. Working solutions were obtained from stock solutions by appropriate dilution in methanol/water solution (70 : 30 *v*/*v*) containing 1 mM ammonium formate to the final concentration of 0.5 *μ*g/mL, 50 *μ*g/mL, and 2 mg/mL of gallic acid, quercetin standards and EAF solutions, respectively. All working standard solutions and samples were filtered through a polyvinylidene fluoride syringe filter (11 mm and 0.45 mm; Millipore Millex, Billerica, MA, USA) before injection into the liquid chromatography coupled to mass spectrometry (LC-MS).

#### 2.6.2. LC-MS Instrumentation and Conditions

LC-ESI-MS analysis was performed using an Agilent 1200 RRHT system (Wilmington, NC, USA) that consisted of a G1311A binary pump, G1379A degasser, and G1316A column oven. These were connected with a CTC sample manager (model 2777, Waters, Milford, CT, USA). The system was coupled to an Applied Biosystems MDS Sciex API 3200 triple quadrupole mass spectrometer (Toronto, Canada) equipped with a syringe pump Harvard 22 Dual Model (Harvard Apparatus, South Natick, MA, USA) and an electrospray ionization (ESI) source. The ESI source was operated in the negative ion mode for quercetin and gallic acid standards monitoring in the ethyl acetate fraction. For the negative ion mode, the mobile phase consisted of methanol/water solution (10 : 90 *v*/*v*) (A) and acetonitrile (B) both containing 1 mM ammonium formate. Analyte separations were carried out according to method established by Oliveira et al. [10]. Chromatographic analysis was performed on an XBridge C18 150 × 2.1 mm (5 mm particle size) column coupled with an XBridge C18 10 × 2.1 mm (5 mm particle size) guard column. The injection volume was 20 *μ*L, and the column temperature was maintained at 25°C. Data acquisition was achieved with the MS Workstation by Analyst 1.4 software (ABI/Sciex). The high-purity nitrogen and zero grade air that were used as the CUR, GS1, and GS2 gases were produced by a high-purity nitrogen generator from PEAK Scientific Instruments (Chicago, IL, USA).

### 2.7. Animal Studies

#### 2.7.1. Experimental Design

Animals were segregated in 6 groups (I to VI) (*n* = 10 each group) and undergone chronic treatment for 90 days. Treatment solutions were administered through gavage (0.1 mL/10 g). Treatment I was designed as a control group (vehicle-distilled water); therefore, only water was administered, while treatments II to VI were test groups; henceforth, AlCl_3_ solution (100 mg/kg) was administered, on the morning, from day 0 to day 90. After the 45th day, a second treatment was orally administered in the afternoon. The second treatment consisted of distilled water (groups I and III), quercetin 30 mg/kg (II), CHE 10 mg/kg (IV), CHE 100 mg/kg (V), and CHE 300 mg/kg (VI). After the treatment period, behavior was evaluated (memory and locomotor activity) and then the animals were sacrificed by cervical dislocation and total brain was removed and stored at 4°C for biochemical and histopathological assays.

#### 2.7.2. Behavioral Studies

Twenty-four hours after the end of the treatment period (91° day), in order to assess the neuroprotective properties of CHE against aluminium-induced neurotoxicity, three behavioral tests were conducted, namely, the step-down test to evaluate short- and long-term memories [16] and open field and chimney tests to evaluate locomotor activity [17, 18].

#### 2.7.3. Biochemical Assays

Twenty-four hours after the last behavioral test, the animals were anesthetized with isoflurane. Subsequently, mice were euthanized by blood extraction through cardiac puncture and the cerebral tissue was removed. Animal's whole brains were immersed in phosphate buffer solution pH 7.4 at a proportion of 1 : 5 *w*/*v*. Dispersion was homogenized in tissue homogenizer (Homo Mix). The resulting colloid was centrifuged at 4492 × g for 20 minutes at 4°C, and the supernatant (biological sample) was assessed on its protein content by Bradford method [19]. Thereafter, the supernatant was also used to assess thiobarbituric acid-reactive species (TBARS) [20], acetylcholinesterase (AChE), and butyrylcholinesterase (BChE) activities [12].

#### 2.7.4. Histopathological Analysis

Animal's cortices were fixed in methanol/chloroform/acetic acid solution (6 : 3 : 1) and then dehydrated with increasing concentrations of ethanol. The dehydrated material was clarified with xylol and embedded in Paraplast (Histosec, Merck). After inclusion, the material was sectioned at 6 *μ*m and stained by hematoxylin-eosin method. Histological sections were examined and digitized using a Zeiss Axio Scope A1 light microscope (Zeiss, Germany). The frontal cortex sections were submitted to morphometric analysis. We quantified the number of viable neurons and the percentage (%) of necrotic eosinophilic neurons per photomicrography (20 fields/group; 40x objective magnification). All analyses were performed using Image Pro-Plus program version 6.1 (Media Cybernetics Inc., Silver Spring, MD, USA). Values were presented as arithmetic mean ± standard deviation of the mean.

### 2.8. Statistical Analysis

Values of IC_50_ were expressed as mean ± the standard error of the mean (SEM) and were obtained by construction of concentration-effect curves (1–500 *μ*g/mL) of three experiments in triplicate using linear regression analysis. Statistical significance was determined using Student's *t*-test or one-way analysis of variance (ANOVA) followed by Tukey's post hoc test, when appropriate. AP value of <0.05 was considered statistically significant. Analyses were performed using GraphPad Prism version 5.00 for Windows (San Diego, CA, USA).

## 3. Results and Discussion

### 3.1. *In Vitro* Anticholinesterase Activity

**Figure 1:**
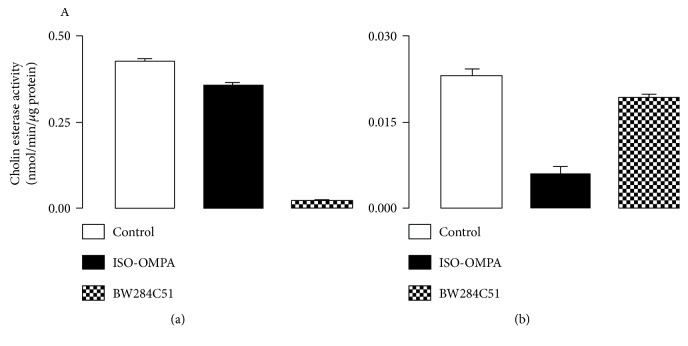
Determination of cholinesterase activity (nmol/min/*μ*g protein) in mice brain homogenate using acetylthiocholine 1.5 mM (a) and butyrylthiocholine 1.5 mM (b) as enzyme substrates and in the presence of iso-OMPA 10 *μ*M (an inhibitor of butyrylcholinesterase) or BW284c51 10 *μ*M (an inhibitor of acetylcholinesterase). Control samples represent the total cholinesterase activity obtained in the absence of enzyme inhibitors. Data were obtained as a mean ± epm of 3 different samples in triplicate.

In order to cross-check whether all cholinesterase activities are fully blocked, control tests were performed for the AChE and BChE activities in mice brain samples incubated with iso-OMPA (an inhibitor of butyrylcholinesterase) or BW284c51 (an inhibitor of acetylcholinesterase) and using ATC or BTC as substrates (Figure 1).

The CHE inhibited the AChE and BChE activities, showing IC_50_ of 202.4 ± 7.9 *μ*g/mL and 204.3 ± 12.4 *μ*g/mL, respectively. The EAF and BF inhibited the enzymes with IC_50_ values of 81.5 ± 14.7 *μ*g/mL and 235.9 ± 17.4 *μ*g/mL for AChE and 118.6 ± 11.3 *μ*g/mL and 225.7 ± 15.2 *μ*g/mL for BChE, respectively. The HF and CF did not show an in vitro anticholinesterase effect (Table 1).

Considering that BChE hydrolyzes ATC, we performed assays using ATC as substrate in the presence of iso-OMPA (an inhibitor of BChE) and the results were not different when compared with those without iso-OMPA (Table 1). Neostigmine, herein used as standard, inhibited AChE and BChE, with IC_50_ values of 88 ± 19.7 ng/mL and 752.5 ± 121 ng/mL, respectively.

**Figure 2:**
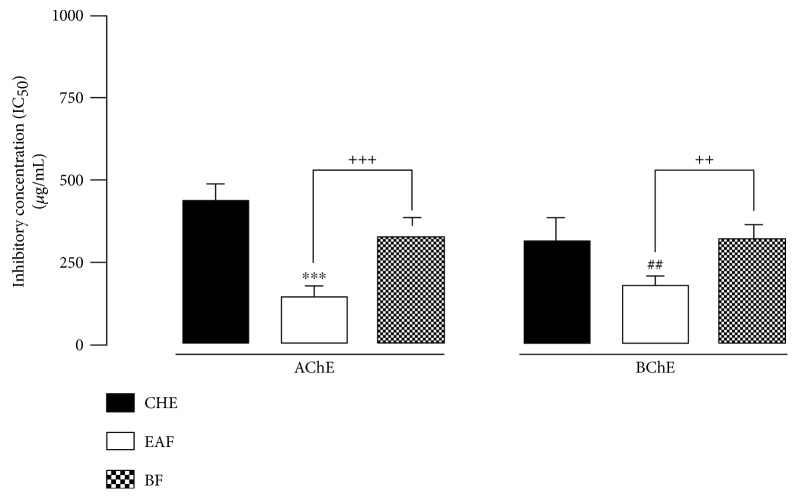
Representative figure of IC_50_ of crude hydroalcoholic extract (CHE), ethyl acetate fraction (EAF), and butanol fraction (BF) of *Caryocar brasiliense* leaves on mice brain acetylcholinesterase and butyrylcholinesterase activities of 3 experiments in triplicate. ^∗∗∗^*P* < 0.001 or ^##^*P* < 0.01 when compared to CHE; ^++^*P* < 0.01 or ^+++^*P* < 0.001 when compared to EAF.

Acetylcholine is responsible for cholinergic neurotransmission, released by nervous presynaptic terminations, and it is the agonist of nicotinic and muscarinic receptors. Normally, AChE rapidly degrades ACh, ending its cellular action [20, 21]. In addition to AChE, another enzyme related to ACh degradation is the BChE enzyme [12]. It has been shown that, in AD, brain BChE activity increases progressively as the severity of dementia progresses, while the AChE activity decreases [2]. In this study, the results showed that CHE, EAF, and BF inhibited both in vitro cholinesterase enzymes, without preferential or selective actions between these two enzymes. On other hand, EAF showed a better cholinesterase inhibitory effect, suggesting that this fraction concentrates the anticholinesterase compounds present in *C. brasiliense* leaves (Figure 2).

### 3.2. Antioxidant Assays

#### 3.2.1. DPPH Assay

CHE inhibited the DPPH oxidation, showing IC_50_ values of 4.6 ± 1.0 *μ*g/mL. HF, CF, EAF, and BF inhibited radical formation with values of IC_50_ of 25.6 ± 1.5 *μ*g/mL, 76.4 ± 0.4 *μ*g/mL, 5.9 ± 0.2 *μ*g/mL, and 9.3 ± 0.6 *μ*g/mL, respectively (Table 1). Quercetin inhibited DPPH oxidation, with an IC_50_ value of 1.46 ± 0.2 *μ*g/mL.

#### 3.2.2. Voltammetric Determination of Redox Behavior

The EI values obtained for the samples studied were 46.6 *μ*A/V (CHE), 27.3 *μ*A/V (HF), 4.4 *μ*A/V (CF), 119.8 *μ*A/V (EAF), and 58.1 *μ*A/V (BF) (Table 1). The order of EI values corresponded to EAF > BF > CHE > HF > CF, indicating that polar fractions showed higher antioxidant potentials. This result agrees with the DPPH results, where the EAF and BF were the fractions that showed higher antioxidant activity.

**Figure 3:**
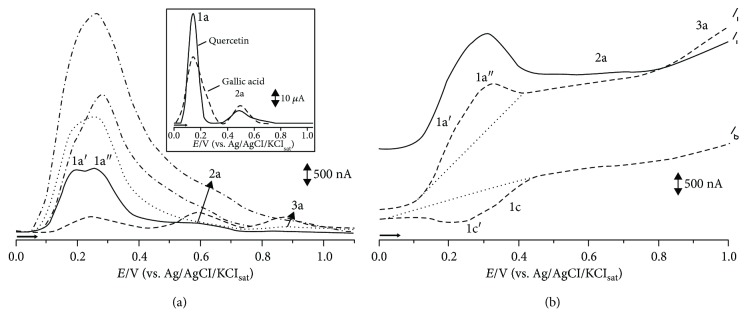
(a) DP voltammograms obtained for of EAF, BF, CHE, HF, and CF extracts. Inset: DP voltammetry of quercetin and gallic acid standards; (b) SWV voltammogram obtained for CHE in pH 7.0 0.1 M phosphate buffer total current (*I*_t_: solid line), forward current (*I*_f_: dashed line) and backward current (*I*_b_: dotted line).

Figure 3(a) shows the DPV voltammograms obtained for CHE and OFs in a 0.1 M phosphate buffer with a pH of 7.0. The DPV voltammogram shows three consecutive oxidation peaks: 1a, at *E*_p1a_≅+0.26 V; 2a, at *E*_p2a_≅+0.59 V; and 3a, at *E*_p3a_≅+0.87 V. These peaks were present in all analyzed samples. In the case of complex samples, such as plant extracts, the formation of current peaks can be the result of contribution of one or more electroactive species in that redox process occurring at similar potentials. This affirmation is illustrated by the peak 2a observed for CHE, EAF, and BF or by peak broadening observed for the other fractions (HF and CF).

In order to establish a better correlation between the aforementioned peaks and the redox profile of potential phytocompounds to which they could be related, a DP voltammetry was conducted with the same standards used in MS analysis, namely, quercetin and gallic acid (Figure 3(a), inset). The peaks 1a at *E*_p1a_≅+0.18 V and 2a at *E*_p2a_≅+0.5 V present in both quercetin and gallic acid are correlated to the redox process that were also observed in all extracts (Figure 3(a)), which is correlated to catechol moiety present in a myriad of natural phenolic compounds with recognized antioxidant performance [18].

Figure 3(b) shows the SWV voltammogram obtained for CHE in the 0.1 M phosphate buffer with a pH of 7.0. The continuous line corresponds to the total current (*I*_t_), which is the sum of the currents related to the oxidation (*I*_f_, forward current) and reduction processes (*I*_b_, backward current), represented by dotted lines. The similarity between the 1a and 1c peaks indicates the reversibility of the system. In the case of antioxidants, the reversibility of a redox process is particularly useful as it relates to its stability and ability to restore the involved species.

### 3.3. Phenolic Content

The levels of phenols were 1.2 ± 0.3 (CHE), 0.6 ± 0.1 (HF), 0.1 ± 0.1 (CF), 4.0 ± 0.2 (EAF), and 1.9 ± 0.3 (BF), where all units are in mg·GAE/mL (Table 1). The highest concentrations of phenolic content were in EAF and BF, fractions that present anticholinesterase and antioxidant activities. It is well established that phenolic compounds, such as flavonoids, are antioxidants and some of them exhibit anticholinesterase activity [13–15]. Therefore, it is possible to suggest that the antioxidant and anticholinesterase activities herein observed are associated, at least in part, to the phenolic compounds.

Ethyl acetate and butanol are higher polarity solvents and extract phenolic compounds (i.e., flavonoids), among other chemical substances [10, 22]. In a previous study, we showed the presence of gallic acid and quercetin in *C. brasiliense* leaves using LC-ESI-MS analysis [10]. In addition to being a well-known antioxidant agent [23], quercetin shows inhibitory activity against AChE and BChE [14, 24] and it was suggested that it may provide a promising approach for the treatment of AD and other oxidative stress-related neurodegenerative diseases [25].

Concerning gallic acid, despite it being an antioxidant agent [26], the anticholinesterase effect is nonsignificant [14]. Regarding these approaches, the presence of quercetin in *C. brasiliense* leaves can shed light on the anticholinesterase and antioxidant effects of *C. brasiliense*. However, further studies should be conducted in order to identify other possible compounds present in the polar OFs of *C. brasiliense* leaves that possess both effects. Furthermore, the differences between the results of colorimetric (DDPH and ABTS) and electrochemical methods can be attributed to the higher selectivity of electroanalytical-based assays, since the color of the samples does influence the readings taken in the spectrophotometer [27, 28].

### 3.4. LC-ESI-MS Analysis

LC-ESI-MS analysis was performed in order to confirm the presence of gallic acid and quercetin in ethyl acetate fraction (EAF) from leaves of *C. brasiliense*. Since EAF and BF fractions originated from CHE, the presence of the markers is nonetheless stated to both extracts. We showed in a previous study the vasorelaxant effects of BF, which were associated with the presence of polyphenols such as gallic acid and quercetin in *C. brasiliense* leaves [10]. These compounds are also described as antioxidant agents, and quercetin presents moreover anticholinesterase effects too. Henceforth, these two compounds were herein selected for a preliminary chromatographic fingerprint analysis of EAF, because this fraction presented the best *in vitro* antioxidant and anticholinesterase effects.

**Figure 4:**
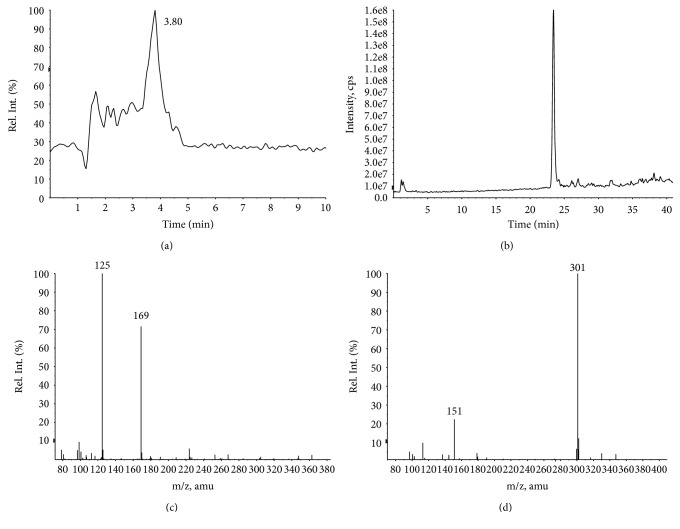
LC-ESI(-)-MS analysis of gallic acid and quercetin standards. Total ion chromatogram of standards: (a) gallic acid, (b) quercetin, (c) MS spectrum of peak extracted in *R*_*t*_ = 3.8 min of gallic acid standard, and (d) MS spectrum of peak extracted in *R*_*t*_ = 23.8 min of quercetin standard.

**Figure 5:**
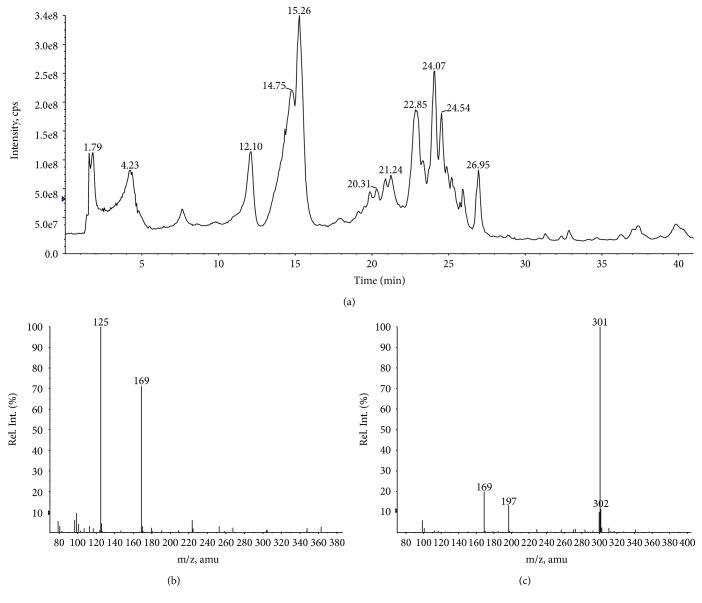
LC-ESI(-)-MS analysis for ethyl acetate fraction (EAF) from leaves of *Caryocar brasiliense*. (a) Total ion chromatogram of EAF, (b) MS spectrum of peak extracted in *R*_*t*_ = 3.8 min, and (c) MS spectrum of peak extracted in *R*_*t*_ = 23.8 min.

The LC-ESI-MS analysis of EAF was realized in the negative mode to confirm the presence of the gallic acid and quercetin, comparing the results with those obtained from the spectra of the standard substances under the same analysis conditions (Figures 4 and 5). The MS spectrum obtained for total ion chromatogram of the EAF sample shows that *R*_*t*_ = 3.8 min (Figure 5) and *R*_*t*_ = 23.8 min (Figures 5(b) and 4(c)). These results prove the presence of gallic acid and quercetin (Figures 4(c) and 4(d)) and are consistent with previously data reported in the literature [29]. In addition, the characteristic of the ion fragment at *m/z* 125 [M–H − CO_2_]^−^, that results of the fragmentation in ESI source of molecular ion [M − H]^−^, *m/z* 169, was observed for both EAF and gallic acid standard in MS spectra. Therefore, the presence of these powerful antioxidants (acid galic and quercetin) in the leaves of *C. brasiliense* is irrefutable.

### 3.5. Behavioral Studies

Owing to the remarkable antioxidant capacity and promising AChE and BChE inhibitor activities of CHE, FB, and EAF extracts, *in vivo* behavioral tests were performed to elucidate the potentialities of this potential herbal candidate to treat memory impairment disorders.

Although EAF exhibited best *in vitro* effects, we used CHE in this step because it was available in the lab in the amount required to treat the animals in all periods of the study. Since behavioral studies allow the assessment of aspects regarding memory retention and locomotor and exploratory capabilities, tests concerning these parameters were conducted. Therefore, behavior was studied with step-down, open field, and chimney tests.

**Figure 6:**
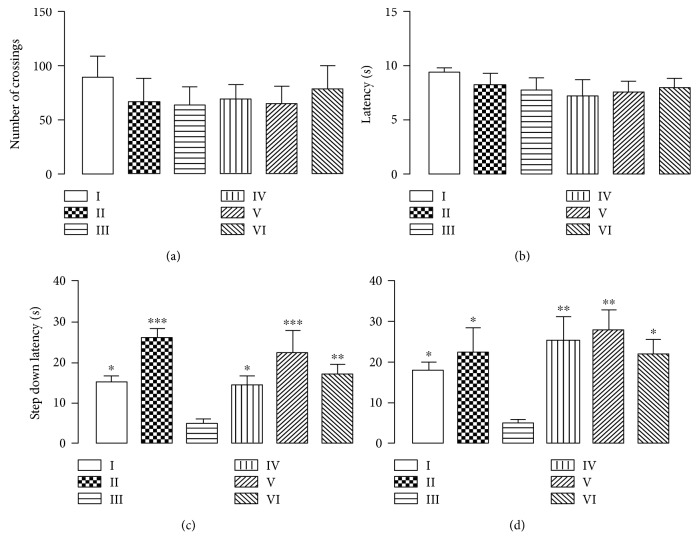
Effect of CHE treatment on locomotor activity (a, b) and memory (c, d) of mice subjected to 90 days of aluminium exposure. (a) Number of crossings of mice groups as evaluated in the open-field test. (b) Time (s) to climb backwards out of the tube within 30 sec for the examined animals in the chimney test. Latencies of retention time (s) in mice as evaluated in the step-down test at 90 min (c) and 24 h (d) after shock challenge. Each column represents mean ± SEM of 10 animals. (^∗^*P* < 0.05, ^∗∗^*P* < 0.01, and ^∗∗∗^*P* < 0.001 in comparison to group III). I: control group; II: quercetin 30 mg/kg; III: aluminium group; IV: CHE 10 mg/kg; V: CHE 100 mg/kg; VI: CHE 300 mg/kg.

Results indicate that CHE promotes memory retention without impairing locomotion (Figure 6). The aluminium-treated group presented both short- and long-term memory impairments, while the control group presented results akin to the literature. However, both the quercetin- and extract-treated groups (IV to VI) presented better memory retention, which implies that CHE do indeed promote neuroprotection and somehow improve murine memory retention. Literature reports that flavonoids exert neuroprotective activities mainly due to their ROS scavenging potential, which corroborates to the results seen in both LC-ESI-MS analysis and the aforementioned tests [22, 23].

### 3.6. TBAR Evaluation

It is established that aluminium neurotoxicity involves oxidative stress and neurodegeneration and that polyphenolic compounds, such as quercetin, attenuate neuronal death against aluminium-induced neurodegeneration [30].

**Figure 7:**
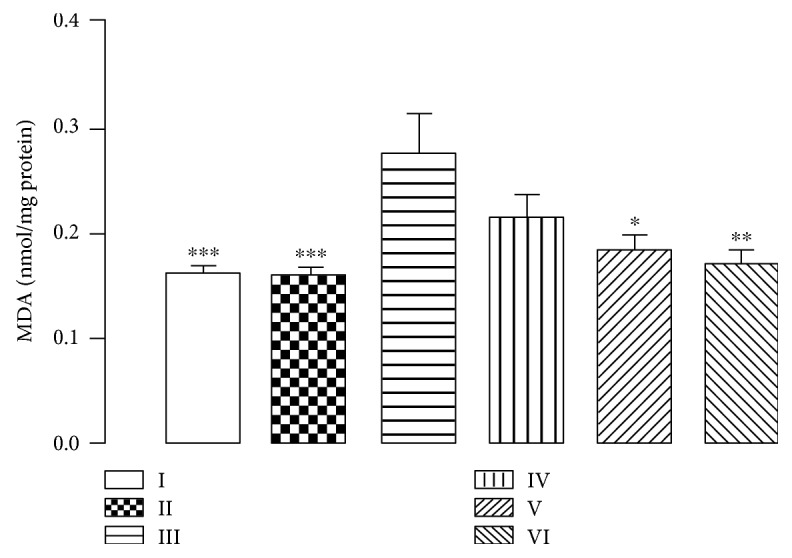
Effect of CHE treatment on malondialdehyde (MDA) concentration in animal whole brains of mice subjected to 90 days of aluminium exposure and treated with CHE (10, 100, and 300 mg/kg). Each column represents mean ± SEM of 10 animals. (^∗^*P* < 0.05, ^∗∗^*P* < 0.01, and ^∗∗∗^*P* < 0.001 in comparison to group III). I: control group; II: quercetin 30 mg/kg; III: aluminium group; IV: CHE 10 mg/kg; V: CHE 100 mg/kg; VI: CHE 300 mg/kg.

Knowing that lipid peroxidation is one of the main manifestations of oxidative damage, we evaluated the CHE protective effect on mice brain cells measuring thiobarbituric acid reactive substances.  Figure 7 shows the CHE-exerted protective effect against neuronal damage promoted by aluminium by minimizing lipid peroxidation. This result is a clear indicator of the extract antioxidant power, which promotes ROS reduction and therefore is implicated in lipid protection against oxidative damage.

### 3.7. Determination of Brain AChE and BChE Activities in Mice Treated with CHE

**Figure 8:**
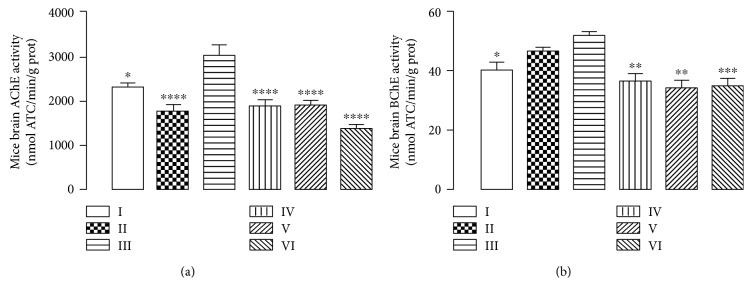
(a) Measure of acetylcholinesterase (AChE) and (b) butyrylcholinesterase (BChE) activities in mice whole brain subjected to 90 days of aluminium exposure and treated with CHE (10, 100, and 300 mg/kg). Each column represents mean ± SEM of 10 animals. (^∗^*P* < 0.05, ^∗∗^*P* < 0.01, and ^∗∗∗^*P* < 0.001 in comparison to group III). I: control group; II: quercetin 30 mg/kg; III: aluminium group; IV: CHE 10 mg/kg; V: CHE 100 mg/kg; VI: CHE 300 mg/kg.

Considering our previous in vitro tests showing the inhibitory effect of pequi leaf extracts on AChE and BChE activities, we aimed to analyze if this effect would be present in animals treated with CHE. In accordance with results of in vitro tests, we observed the CHE inhibitory effect on cholinesterase enzymes in brain tissue of aluminium-intoxicated mice (Figure 8).

Since it is described that the increase of brain acetylcholine levels attenuates memory deficits [31] and, in opposition, aluminium chloride treatment increases the activity of mouse brain cholinesterase [32], we hypothesize that the inhibition of cholinesterase activity by CHE is a mechanism involved in the protection against memory impairment produced by aluminium.

### 3.8. Histopathological and Morphometrical Analyses

**Figure 9:**
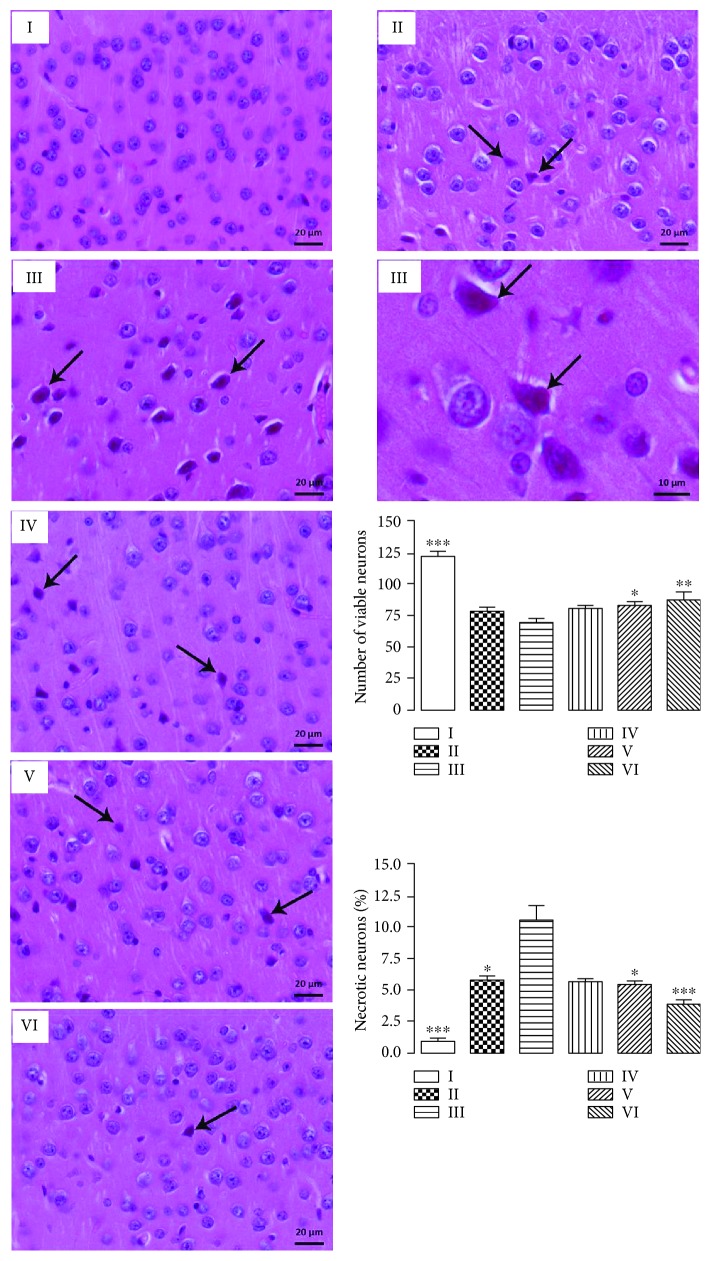
Histological sections of the frontal cerebral cortex stained by hematoxylin-eosin method. I: control group; II: quercetin 30 mg/kg; III: aluminium group; IV: CHE 10 mg/kg; V: CHE 100 mg/kg; VI: CHE 300 mg/kg. All aluminium-exposed groups presented shrunken neurons with cytoplasm being intensely eosinophilic (arrows). These necrotic neurons presented pyknotic nucleus with no discernible nucleolus. In the graphs, each column represents mean ± SEM of 3 animals per group. (^∗^*P* < 0.01, ^∗∗^*P* < 0.001, and ^∗∗∗^*P* < 0.0001 in comparison to group III).

Morphoquantitative data demonstrated that aluminium decreased the number of viable neurons in the cerebral cortex, promoting a high percentage of eosinophilic neuronal necrosis (Figure 9). CHE (V and VI groups) increased the number of viable neurons and decreased the rate of neuronal death (Figure 9). These effects may be correlated to the neuroprotection exerted by phenolic compounds present in CHE, which were nonetheless detected in LC-MS. The results therefore show the potential of pequi leaves in counteracting the damage inflicted by aluminium on mice brain.

## 4. Conclusions

This study reports for the first time the anticholinesterase properties of *C. brasiliense*. The higher polarity fractions of *C. brasiliense* leaves presented high levels of phenols and both antioxidant and anticholinesterase activities. Behavioral tests revealed that pequi leaf extract protects against aluminium-induced memory impairment and inhibits lipid peroxidation and cholinesterase activity. Further histopathological studies revealed that pequi attenuates aluminium-induced cell necrosis, increasing neuronal viability. Taken together, these results indicate that pequi leaves may represent a new approach towards treatments to reverse the neuronal death, in order to slow down the progression of neurodegenerative diseases such as Alzheimer disease.

## Figures and Tables

**Table 1:** Values of IC_50_ for AChE, AChE in the presence of iso-OMPA (10^−5^ M) and BChE activities, and DPPH free radical formation of CHE and OFs (HF, CF, EAF, and BF) of *C. brasiliense* leaves. Values of EI and total phenolic content (GAE) to the samples are listed as well.

	AChE (*μ*g/mL)	AChE + iso-OMPA (*μ*g/mL)	BChE (*μ*g/mL)	DPPH (*μ*g/mL)	EI (*μ*A/V)	GAE (mg/mL)
CHE	202.4 ± 7.9	233.6 ± 12.2	204.3 ± 12.4	4.6 ± 1.0	46.6 ± 3.9	1.2 ± 0.3
HF	ND	ND	ND	25.6 ± 1.5^∗∗∗^	27.3 ± 2.5^∗∗^	0.6 ± 0.1
CF	ND	ND	ND	76.4 ± 0.4^∗∗∗^	4.4 ± 0.5^∗∗∗^	0.1 ± 0.1^∗^
EAF	81.5 ± 14.7^∗∗∗^	128.9 ± 16.8^∗∗∗^	118.6 ± 11.3^∗∗∗^	5.9 ± 0.2	119.8 ± 4.0^∗∗∗^	4.0 ± 0.2^∗∗∗^
BF	235.9 ± 17.4	292.5 ± 22.3	225.7 ± 15.2	9.3 ± 0.6^∗^	58.1 ± 2.7	1.9 ± 0.3

Values are expressed as mean ± SEM of three experiments. ^∗^*P* < 0.05, ^∗∗^*P* < 0.01, and ^∗∗∗^*P* < 0.001 when compared with CHE. ND: not determined.

## Data Availability

All data used to support the findings of this study are included within the article.

## Abbreviations

AChE: Acetylcholinesterase
AD: Alzheimer's disease
BChE: Butyrylcholinesterase
BF: Butanol fraction
CF: Chloroform fraction
CHE: Crude hydroalcoholic extract
DPPH: 2,2-Diphenyl-1-picrylhydrazyl
DPV: Differential pulse voltammetry
DTNB: 5,5′-Dithiobis(2-nitrobenzoic acid)
EAF: Ethyl acetate fraction
EI: Electrochemical index
*E*_pa_: Potentials of anodic peaks
ESI: Electrospray ionization
GAE: Gallic acid equivalent
GCE: Glassy carbon electrode
HF: Hexane fraction
*I*_b_: Backward current
*I*_f_: Forward current
*I*_pa_: Currents of anodic peaks
*I*_t_: Total current
LC-MS: Liquid chromatography coupled to mass spectrometry
OFs: Organic fractions
ROS: Reactive oxygen compounds
SEM: Standard error of the mean
SWV: Square wave voltammetry.
